# Asymmetric branched F-actin networks at apical clathrin coated pits support microvillar biogenesis

**DOI:** 10.1091/mbc.E25-12-0606

**Published:** 2026-04-08

**Authors:** Olivia L. Perkins, Malcom Díaz García, Matthew J. Tyska

**Affiliations:** ^a^Department of Cell and Developmental Biology, Vanderbilt University School of Medicine, Nashville, TN 37232; Toronto Metropolitan University

## Abstract

Microvilli are conserved actin-based protrusions that expand surface area and absorptive capacity in epithelial tissues. In humans, the intestinal tract grows 300 trillion microvilli per day, yet the molecular mechanisms defining where and when microvilli grow remain unclear. Using a combination of live-cell confocal and super-resolution microscopy to investigate native intestinal tissues and epithelial cell culture models, we found that microvilli grow from Arp2/3-generated branched actin networks that form on the surface of clathrin-coated pits. These transient networks are stabilized by cortactin and localize to the apical plasma membrane minutes before reorganizing into the linear core actin bundles that support microvilli. Moreover, subpixel precision localization of coated pit and microvilli markers revealed that F-actin asymmetrically localizes to one side of a nascent clathrin-coated pit, and that microvilli grow from the side with the highest F-actin density. These findings support a model where the asymmetric accumulation of F-actin and growing barbed ends on the surface of a clathrin-coated pit offers a filament source to support microvilli formation.

## Abstract (executive summary)

- Microvilli are actin-bundle-supported protrusions that are assembled by solute-transporting epithelial cells to expand apical surface area; how these cells spatially and temporally control microvilli formation remains poorly understood.
- Using live-cell and super-resolution microscopy, we found that microvilli arise from asymmetric Arp2/3-dependent actin networks, which form on the surface of clathrin-coated pits and reorganize into the linear actin bundles that support protrusions.
- These results deepen our understanding of mechanisms that control the formation of cell surface features and may offer insight into the biogenesis of microvilli-like protrusions found in other epithelial systems.

## INTRODUCTION

In solute-transporting tissues, constituent epithelial cells present densely packed arrays of micron-scale surface protrusions, known as microvilli, which collectively form a brush border. These specializations increase apical membrane area 60–120-fold (Helander and Fandriks, 2014; Delacour *et al.*, 2016), in turn maximizing the holding capacity for channels and transporters that are required for physiological function. In epithelial tissues that exhibit continuous regenerative renewal, such as the intestinal tract, microvilli continuously assemble throughout the full life of an organism. In humans, based on the rate of enterocyte turnover (∼10^11^/day; Williams *et al.*, 2015) and the number of microvilli that extend from the average enterocyte (∼3000), the small intestine must grapple with the energetic demands of building ∼3 × 10^14^ new microvilli each day. Despite the central role microvilli play in epithelial function and their continuous assembly under homeostatic conditions, our understanding of mechanisms that promote their growth remains limited.

From previous studies, we know that an individual microvillus is supported by a core actin bundle containing 15–30 filaments, which exhibit unipolar orientation with barbed ends extending out into the distal tip compartment. Filaments in core bundles are linked by espin, villin, fimbrin, and MISP (Mooseker and Tilney, 1975; Bretscher and Weber, 1979, 1980; Bartles *et al.*, 1998; Morales *et al.*, 2022). Membrane-actin crosslinkers such as ezrin and myosin-1a help to stabilize the protrusion and maintain membrane wrapping (Tyska *et al.*, 2005; Liu *et al.*, 2012). Distal tip-localized complexes containing epidermal growth factor pathway substrate 8 (EPS8) and insulin-receptor tyrosine kinase substrate (IRTKS) control barbed-end dynamics, including filament elongation and capping (Croce *et al.*, 2004; Postema *et al.*, 2018; Gaeta *et al.*, 2021). Moreover, several recent studies have begun to explore how these actin-binding factors are recruited to the apical surface to support microvillus assembly. (Meenderink *et al.*, 2019; Gaeta *et al.*, 2021; Gaeta and Tyska, 2023). How microvillar growth sites are specified at the plasma membrane is still poorly understood, although emerging evidence points to apical clathrin-coated pits (CCPs) as key organizational hubs for this activity (Perkins *et al.*, 2025).

Canonically, clathrin-mediated endocytosis (CME) is one pathway that cells use to internalize plasma membrane and membrane-associated proteins (Kaksonen and Roux, 2018). On the apical surface of epithelial cells, force generated by actin polymerization is essential for overcoming the high membrane tension that would otherwise impede CCP invagination and vesicle scission (Hyman *et al.*, 2006; Boulant *et al.*, 2011). The molecular events driving actin polymerization during CME are well described in both yeast and mammalian cells (Kaksonen and Roux, 2018). Briefly, accumulation of phosphatidylinositol 4,5 bisphosphate (PI(4,5)P_2_; Jost *et al.*, 1998) triggers the recruitment of adapter protein-2 (AP-2) to the plasma membrane, which in turn recruits clathrin. PI(4,5)P_2_ accumulation also recruits neuronal-Wiskott-Aldrich syndrome protein (N-WASP; Merrifield *et al.*, 2004), actin-related protein 2/3 complex (Arp2/3; Winter *et al.*, 1997), capping protein (Kim *et al.*, 2006), and actin bundling and severing proteins (Kubler and Riezman, 1993; Idrissi *et al.*, 2002). These proteins work together to drive the polymerization, crosslinking, and turnover of the branched actin network surrounding the CCP (Kaksonen and Roux, 2018).

Based on our recent work, apical CCPs appear to serve as permissive sites for microvillar growth, potentially due to their dense actin networks. Indeed, live confocal imaging showed that apical CCPs and microvilli share molecular resources during a 3–4 min timeframe that overlaps with protrusion growth. Additionally, inhibition of the Arp2/3 complex significantly reduced the growth and accumulation of new microvilli on the cell surface, suggesting that core bundle actin filaments emerge from Arp2/3-generated networks (Perkins *et al.*, 2025). In that study, we also noticed that only a subset of CCPs gives rise to microvilli. This opened questions about which pit features (e.g., molecular composition or physical properties) might favor microvillar assembly. One possibility is that CCPs capable of supporting protrusion growth are distinguished by a specific organization of their associated actin network. While previous studies have shown that actin polymerization is required for CME under conditions with high membrane tension (Hyman *et al.*, 2006; Boulant *et al.*, 2011), it remains unclear how the architecture of the filament network might influence or enable protrusion growth.

Studies in nonepithelial systems suggest a mechanistic framework for how these CCPs might support microvillar growth. Total internal reflection fluorescence microscopy (TIRF) and transmission electron microscopy (TEM) revealed that F-actin and actin-binding proteins can localize asymmetrically around CCPs, rather than forming uniform collars (Collins *et al.*, 2011; Jin *et al.*, 2022). Complementary work using ion conductance microscopy showed that this uneven F-actin distribution drives the formation of an actin-filled membrane bulge adjacent to the pit, in a process referred to as “asymmetric pit closure” (Shevchuk *et al.*, 2012). Notably, such asymmetric actin filament assembly appears to be the predominant mode of pit closure, occurring in ∼70% of events in some cell types (Yu and Yoshimura, 2023). Together, these studies suggest that the default architecture of actin at many CCPs is directionally biased, which gives rise to a localized region of force production and membrane deformation. Asymmetric F-actin topology also offers a compelling explanation for why CCPs serve as productive sites for microvillar growth. In this scenario, the barbed ends of Arp2/3-generated filaments could be arranged to coalesce into a nascent microvillus through a process similar to the “convergent elongation” mechanism that drives filopodial assembly (Vignjevic *et al.*, 2006; Yang and Svitkina, 2011). Understanding which CCPs support protrusion assembly is central to identifying the molecular and physical cues that initiate microvillar formation.

Here, we investigate the organization of actin filaments at the apical CCPs that give rise to microvilli. Using super-resolution light microscopy, we show that these sites are characterized by a dense, branched, Arp2/3-generated actin network, stabilized by cortactin. Intriguingly, the majority of clathrin puncta that were associated with F-actin were also associated with a nascent microvillus. Consistent with previous literature, actin filaments on the surface of these CCPs asymmetrically accumulated on one side, and strikingly, microvilli frequently emerged from the side of the pit where actin is most enriched. Furthermore, CIP4, a known marker of asymmetric pit closure, was present at every microvillus growth event we observed, suggesting a potential mechanism for microvillar assembly. These findings suggest that asymmetric F-actin accumulation defines the subset of CCPs that are competent to initiate microvillar growth.

## RESULTS

### Arp2/3 complexes localize sub-apically in native intestinal epithelial cells

Dense, branched F-actin meshworks are a hallmark of apical CCPs, where Arp2/3-mediated filament assembly supports membrane invagination (Boulant *et al.*, 2011; Lacy *et al.*, 2018). Given that Arp2/3-generated networks can be reorganized into linear bundles to form filopodia through convergent elongation (Yang and Svitkina, 2011), we reasoned that epithelial microvilli might emerge through an analogous filament remodeling process. To test this, we first examined the localization of the Arp2/3 complex subunit ArpC2 in the crypts of native mouse small intestinal tissue. Nascent enterocytes that reside in the crypt are actively differentiating, building new microvilli as they migrate through the crypt-villus transition zone and onto the villus surface. Confocal imaging of stained sections revealed strong subapical enrichment of ArpC2 in both crypt and villus compartments, with a signal that overlapped with the band of γ-actin that marks the terminal web (Figure 1A). Consistent with the higher numbers of microvilli per cell in the villus versus crypt domains, villus: crypt intensity ratios for the subapical γ-actin signal were greater than 1 (Figure 1B). Notably, the ArpC2 signal demonstrated comparable enrichment ratios (Figure 1B). These data indicate that, in vivo, Arp2/3 complexes are found at sites where microvilli grow and demonstrate the enrichment that one would expect for a factor that drives microvillar growth through the crypt-villus transition.

**FIGURE 1:**
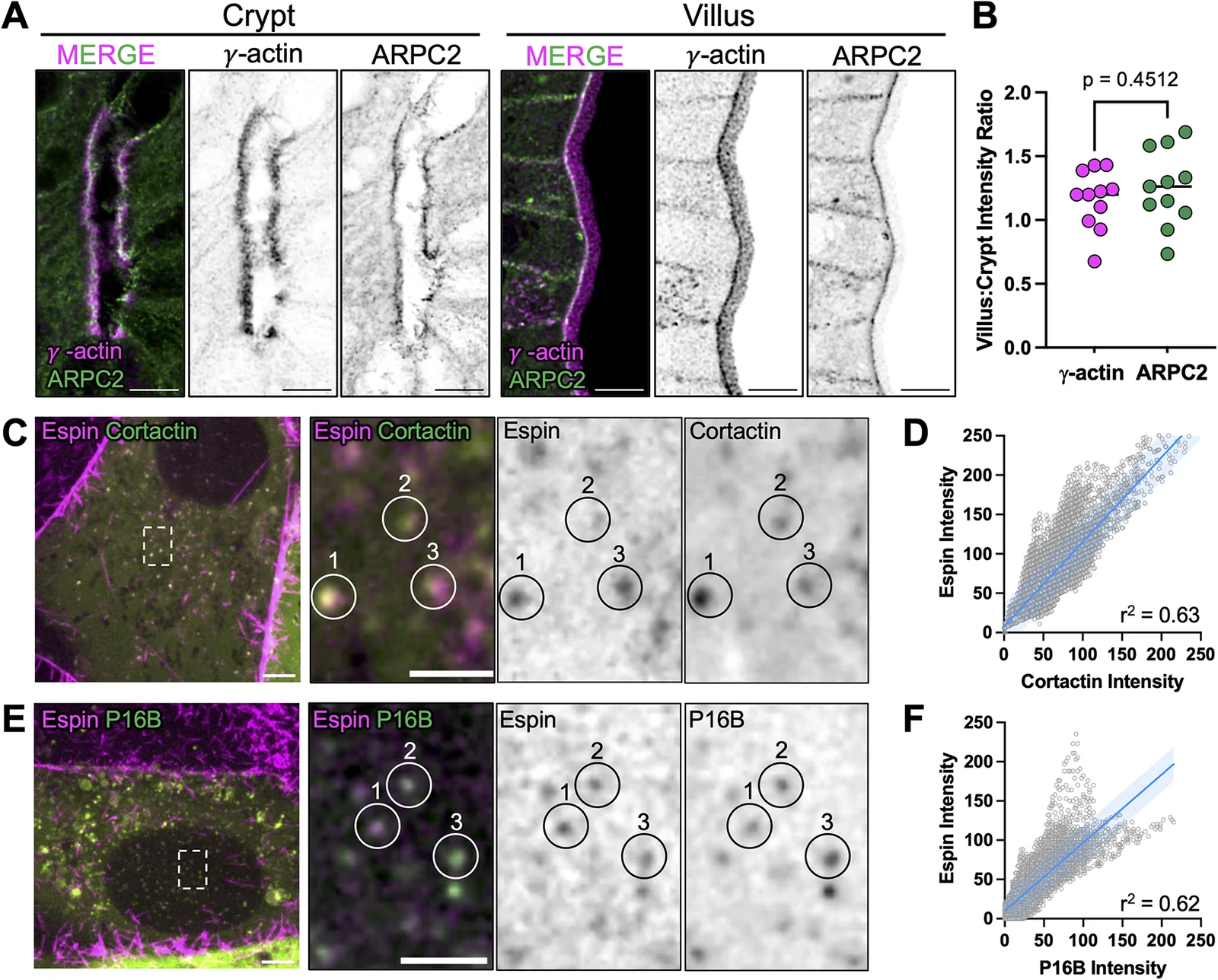
Arp2/3 localization in native intestinal tissues and differentiating epithelial cells in culture. (A) Native mouse intestinal tissue stained for γ-actin (magenta) and ArpC2 (green); images show crypt and villus domains (left and right, respectively). (B) The ratio of fluorescence intensity of γ-actin and ArpC2 in the crypts versus villi; *n* = 11 crypt-villus axes from two mice. (C) Apical surface maximum intensity projection of CL4 cells expressing espin and cortactin. Scale bar = 5 µm. (D) Intensity correlation plot of espin and cortactin signal, generated from the representative images in C. Blue line shows the best linear fit; correlation coefficient is shown on the plot. (E) Apical surface maximum intensity projection of CL4 cell expressing espin and P16B. Scale bar = 5 µm. (F) Intensity correlation plot of espin and P16B signals, generated from the representative images in E. Blue line shows the best linear fit; correlation coefficient is shown on the plot.

### Arp2/3 complexes co-localize with microvillar markers at the apical surface in epithelial cell culture

To further explore the localization of Arp2/3 complexes relative to growing microvilli, we turned to the LLC-PK1-CL4 (CL4) epithelial culture model, which enables visualization of individual protrusions as they grow stochastically, early in differentiation. Co-expression of cortactin, a canonical marker of branched F-actin, with the microvillar-specific actin bundler espin, revealed discrete apical puncta where the two proteins exhibited strong colocalization (Figure 1, C and D). Expression of P16B, an Arp2/3 complex component, further confirmed that espin-marked microvillar precursors overlap with Arp2/3 complexes (Figure 1, E and F). To determine if the assembly of these puncta depends on Arp2/3 activity, we treated cells with Arp2/3 inhibitor CK666 (Nolen *et al.*, 2009). Strikingly, this compound nearly eliminated cortactin puncta (Supplemental Figure S1) across the apical surface, indicating that their formation and/or stability depend on branched F-actin assembly.

### Microvilli grow from Arp2/3-generated branched F-actin networks

If Arp2/3 complexes generate filaments that are eventually remodeled into the core actin bundles that support microvilli, one might expect cortactin and P16B accumulation in apical puncta to precede espin localization to these sites. Indeed, spinning disk confocal microscopy revealed that these puncta were highly dynamic and frequently elongated into mature microvilli (Figure 2, A and C; Supplemental Movie S1). Analysis of intensity over time revealed that cortactin and P16B puncta appeared ∼1–2 min before detectable espin accumulation (Figure 2, B and D), suggesting that transient branched actin networks mark sites of microvillar formation. We also noted that cortactin signal decreased following the accumulation of espin, suggesting a transition from a branched actin network to a linear bundle at these sites (Figure 2, C and D). Together, these data demonstrate that Arp2/3-generated branched actin networks are positioned at the apical surface during the onset of microvillar growth. The subsequent emergence of microvilli from these foci provides evidence that microvillar assembly could proceed through the reorganization of branched actin networks, consistent with a convergent elongation mechanism.

**FIGURE 2:**
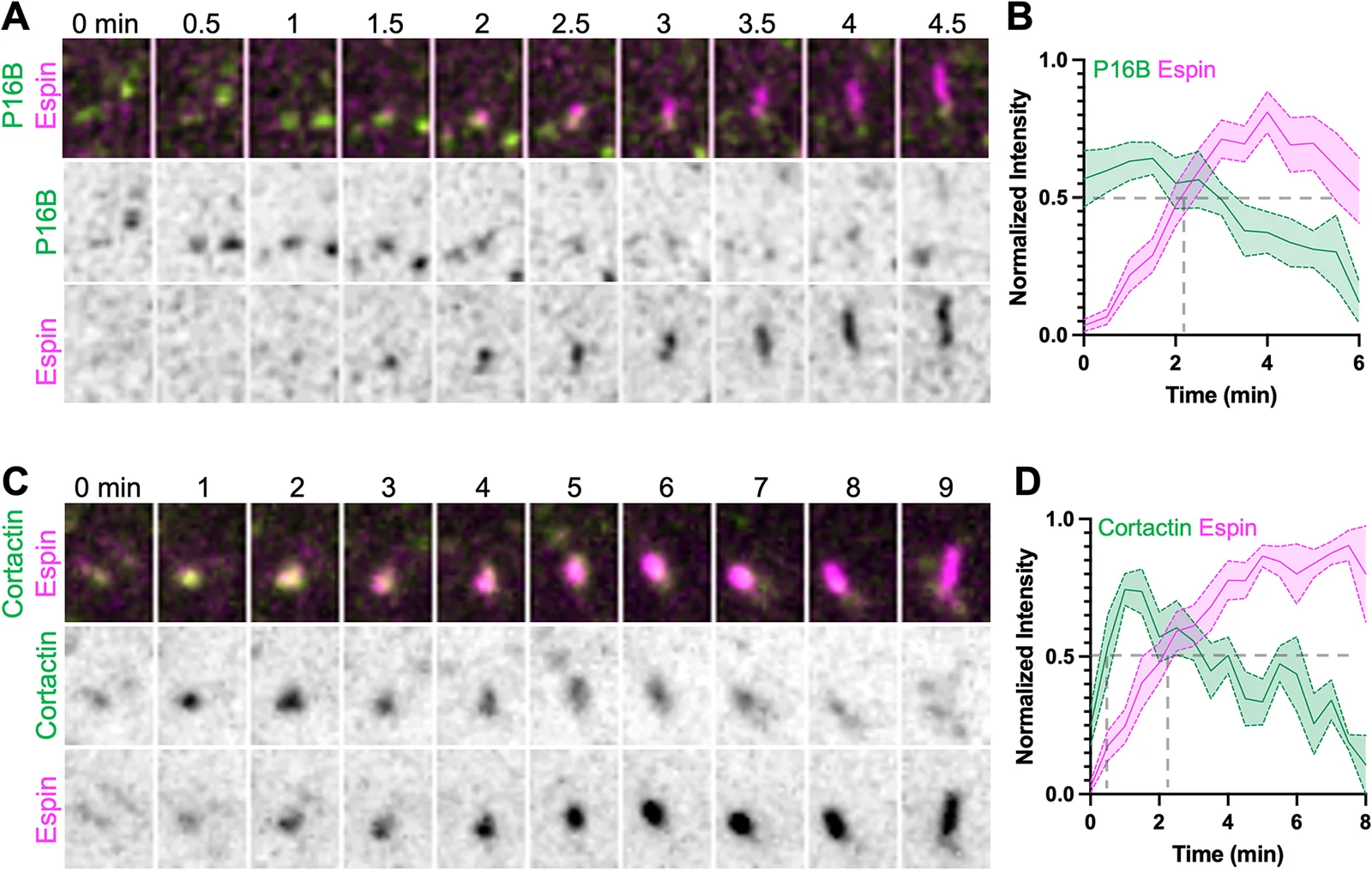
Microvilli grow from Arp2/3-generated branched F-actin networks. (A) Montage showing a single microvillus growth event from a CL4 cell expressing espin (magenta) and P16B (green). Box width = 2.09 µm. (B) Average fluorescence intensity measurements over time; *n* = 11 growth events from four cells and two independent experiments. (C) Montage showing a single microvillus growth event from a CL4 cell expressing espin (magenta) and cortactin (green). Box width = 2.09 µm. (D) Average fluorescence intensity measurements over time; *n* = 11 growth events from four cells across two independent experiments.

### Microvilli grow from clathrin-coated pits exhibiting asymmetric F-actin networks

Having established that branched actin filament networks mark microvillar growth sites, we next asked how these networks are spatially organized on the surface of CCPs. To investigate this, we turned to super-resolution imaging of CL4 cells expressing espin and stained for F-actin and clathrin light chain A (CLTA; Figure 3A). Quantification of punctate signal overlap revealed that ∼61% of CLTA puncta that were associated with F-actin also contained espin, suggesting that the majority of actin-enriched CCPs could serve as microvillar initiation sites (Figure 3B). We next applied a subpixel precision localization analysis to examine the relative positions of marker signals in individual puncta. The coordinates of each marker punctum were then used to calculate pairwise distances between signals at sites of microvillar growth. The mean nearest-neighbor distance between F-actin and clathrin signals was ∼226 nm (Figure 3C), which is consistent with previous ultrastructural measurements in CL4 cells that placed apical endocytic pits within ∼250 nm of nascent microvilli (Perkins *et al.*, 2025). Moreover, inspection of individual puncta revealed that F-actin networks were not symmetrically arranged around CCPs (Figure 3D). Pits lacking espin exhibited diffuse, but asymmetric actin accumulation, whereas pits associated with nascent microvilli (marked by espin) displayed sharply asymmetric actin densities concentrated on one side of the pit (Figure 3D). Quantification of actin intensity profiles showed only a modest increase in total actin intensity around CCPs that had initiated microvilli (Figure 3E), suggesting that microvillar growth is primarily driven by the reorganization of actin filaments rather than the *de novo* growth of actin filaments. To quantify the distribution of F-actin surrounding the CCP, we calculated a radial concentration parameter using the center of the CCP as a spatial point of reference. Briefly, radial concentration was calculated as an intensity-weighted mean (R), where higher values indicate a higher concentration of signal around a specific angle (i.e., higher asymmetry). Radial concentration analysis revealed a significant increase in F-actin asymmetry at CCPs associated with microvilli (Figure 3F). Together, these findings reveal that the branched F-actin networks from which microvilli grow are not uniformly organized around the apical CCPs. Instead, actin filaments are asymmetrically distributed, with the microvilli forming on the side of the pit with the highest F-actin density.

**FIGURE 3:**
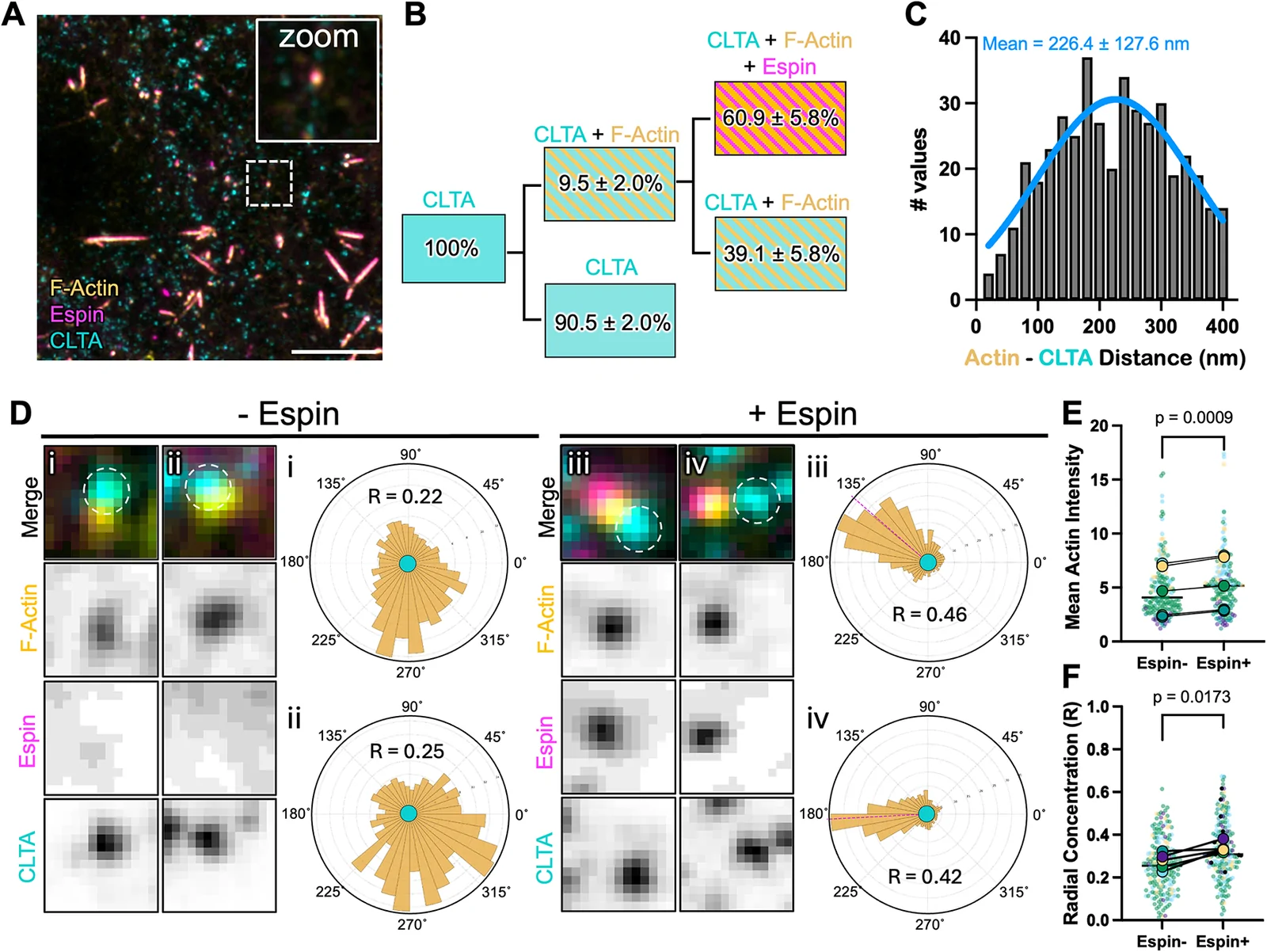
Microvilli grow from clathrin-coated pits with asymmetric F-actin. (A) Maximum intensity projected super resolution image showing a CL4 cell expressing espin (magenta) and stained for CLTA (cyan) and F-actin (yellow). Scale bar = 5 µm. (B) Population analysis of the association of punctate actin and espin signals with CLTA foci; *n* = 7441 CLTA puncta from five cells. (C) Histogram of distances between phalloidin and CLTA centroids. Blue line shows a Gaussian fit; *n* = 428 spots from five cells across two independent experiments. (D) Individual puncta showing actin-associated CLTA foci with and without espin; associated polar plots show actin intensity measured in a 400 nm radius surrounding the CLTA punctum (cyan). All image boxes are 750 nm wide. (E) Mean actin intensity from puncta with versus without espin; *n* = 197 without espin, *n* = 188 with espin. Small transparent spots represent individual pits, and large opaque circles show individual cell means. (F) Radial concentration (R) of the actin signal at puncta with versus without espin. Radial concentration was calculated as the intensity-weighted mean (R), where higher values indicate sharper angular localization. Small transparent spots represent individual pits, large opaque circles show individual cell means; *n* = 197 without espin puncta, *n* = 188 puncta with espin.

### CIP4, a marker of asymmetric CCP closure, labels sites of microvillar growth

Asymmetric F-actin accumulation on a CCP is linked to the formation of a membrane “bulge” or “cap,” which extends over the pit opening to drive asymmetric closure (Shevchuk *et al.*, 2012; Yu and Yoshimura, 2023). In these cases, application of asymmetric stresses to the membrane may reduce the energy cost for pit closure and accelerate membrane fission (Vasan *et al.*, 2020; Jin *et al.*, 2022). Previous studies revealed that the F-BAR protein Cdc42-interacting protein (CIP4, also known as TRIP10) marks asymmetric pit closure sites in nonepithelial cells (Yu and Yoshimura, 2023). From this perspective, we sought to determine if the apical CCPs that demonstrate asymmetric F-actin enrichment and colocalization with espin are marked by CIP4. To test this idea, we expressed CIP4 in CL4 cells, stained for F-actin and CLTA, and then performed super-resolution microscopy (Figure 4A). Analysis of punctate signal overlap revealed that ∼52% of CLTA puncta were associated with CIP4 (Figure 4B). Interestingly, of these CIP4-positive pits, ∼16% exhibited F-actin enrichment (Figure 4B). Consistent with previous studies, CIP4-labeled CCPs displayed asymmetric accumulation of F-actin relative to the center of the clathrin punctum (Figure 4C). We next asked whether these structures represent sites of microvillar growth. To directly visualize the temporal relationship between CIP4 recruitment and microvillar growth, we once again performed live imaging of differentiating CL4 cells expressing marker proteins. Time-lapse montages showed that microvilli, marked by espin, emerge from CIP4 puncta (Figure 4, D and E). Moreover, CIP4 localized in puncta at the apical surface ∼2 min before detectable espin accumulation and elongation, a timeframe consistent with the arrival of branched actin filaments (marked by cortactin and P16B, Figure 2). Together, these results demonstrate that sites of asymmetric pit closure, marked by CIP4 and asymmetric F-actin assembly, are primed to grow microvilli.

**FIGURE 4:**
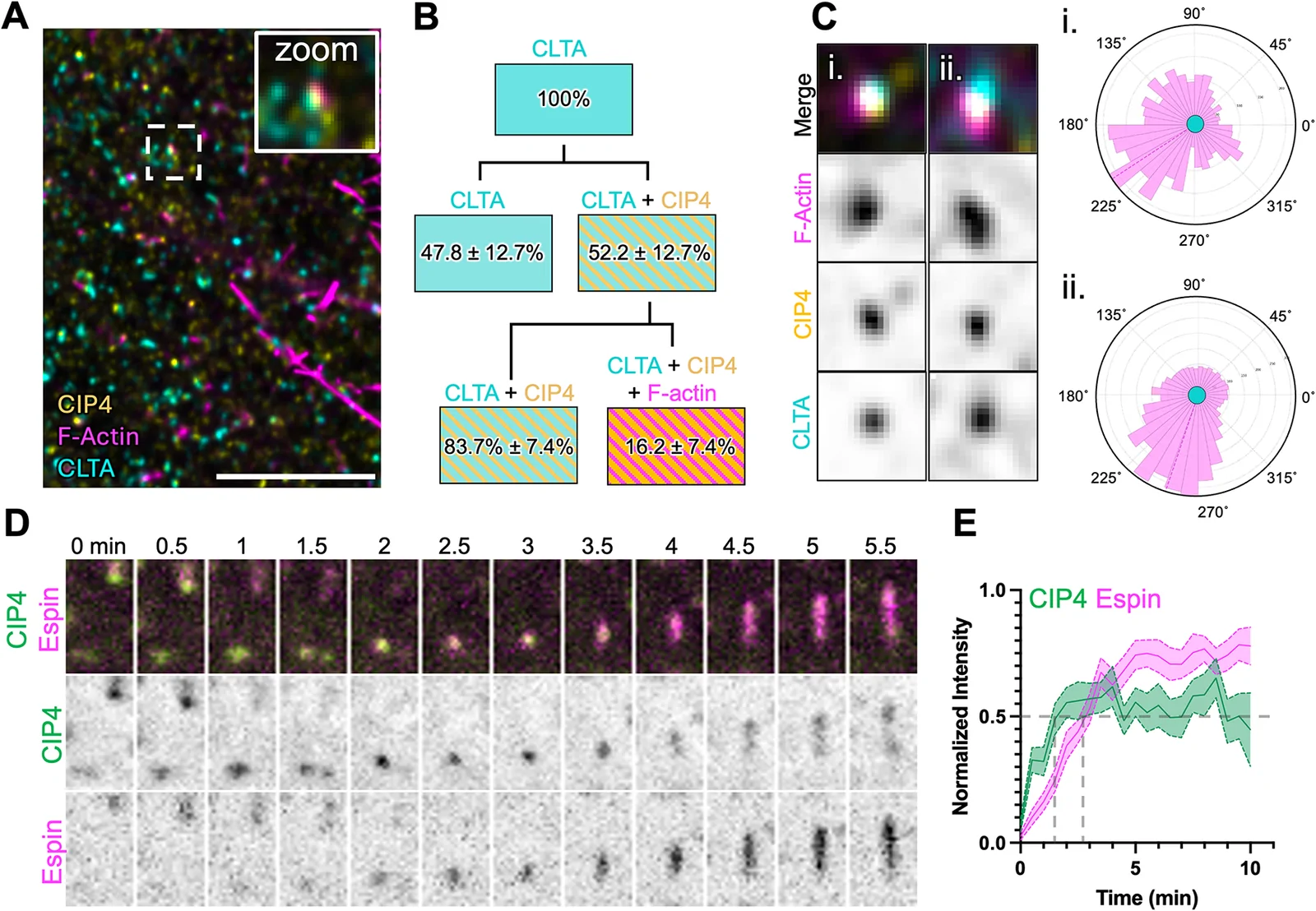
CIP4 marks clathrin-coated pits that support microvillar growth. (A) N-SPARC maximum intensity projection image of a CL4 cell expressing espin and CIP4, and stained for actin and CLTA. Scale bar = 5 µm. (B) Population analysis showing the association of CIP4 and actin with CLTA foci; *n* = 3358 CLTA puncta from four cells across two independent experiments. (C) Representative individual puncta showing CIP4, actin-associated CLTA foci. Associated polar plots show F-actin intensity measured in a 400 nm radius surrounding the CLTA punctum. All image boxes are 750 nm wide. (D) Montage of a single microvillus growth event in a CL4 cell expressing espin and CIP4. (E) Average fluorescence intensity measurements over time; *n* = 16 growth events from six cells across three independent experiments.

## DISCUSSION

Recent work from our group established that microvillar biogenesis is mechanistically linked to clathrin-mediated endocytosis (Perkins *et al.*, 2025). However, live imaging experiments in that study showed that only a subset of CCPs gives rise to microvilli, leading to a question about what distinguishes this population of CCPs. In the current study, we sought to further define the characteristics of CCPs that support microvillar growth. Using super-resolution imaging of fixed epithelial cells and live confocal imaging of actively differentiating cells, we observed that microvilli emerge from CCPs featuring dense, F-actin networks that are asymmetrically positioned relative to the center of the pit. The asymmetric positioning of F-actin on a large fraction of pits, as indicated by the distribution of radial concentration values, is consistent with previous studies on CCP architecture (Shevchuk *et al.*, 2012; Yu and Yoshimura, 2023). Importantly, because our super-resolution datasets represent steady-state snapshots of an asynchronous population of endocytic events, the CCPs sampled here represent various stages of maturation. We propose that the large population of actin-positive CCPs that also had detectable espin signal represents microvillus precursors. Live-cell microscopy reinforced this interpretation as actin-rich foci elongated into mature microvilli in minutes. The strong association between the Espin signal and actin-associated CCPs supports a model where a critical determinant for whether a CCP grows a microvillus is the asymmetric accumulation of F-actin.

How are CCP-associated branched actin networks reorganized into the parallel core bundles that support microvilli? Previous studies on filopodial assembly led to the convergent elongation model, where a subset of filaments that originate in the meshwork of the lamellipodium are protected from capping, sequestered by actin bundlers, and then permitted to elongate to support a filopodium (Vignjevic *et al.*, 2006; Yang and Svitkina, 2011). In that system, Ena/VASP provides anti-capping activity and promotes filament elongation (Schirenbeck *et al.*, 2005; Mellor, 2010; Yang and Svitkina, 2011). Although this factor is not enriched in the intestinal brush border (McConnell *et al.*, 2011), other tip-targeting residents of microvilli might bring similar activities to promote persistent elongation of filament barbed ends. Candidates include Eps8 and IRTKS, which both impact barbed-end dynamics and mediate membrane-cytoskeleton interactions; they also localize apically, precisely at the onset of microvillar growth (Postema *et al.*, 2018; Gaeta *et al.*, 2021). Based on recent CryoEM studies, CCP-associated barbed ends are aligned to generate force against the membrane (Serwas *et al.*, 2022). The asymmetric accumulation of F-actin observed here and in earlier studies (Jin *et al.*, 2022) might spatially concentrate force-generating barbed ends and reduce the distance between neighboring filaments to within the effective range of core bundlers villin, espin, fimbrin, and MISP (maximum reach ∼10–15 nm; Matsudaira *et al.*, 1983; Bartles *et al.*, 1998; Volkmann *et al.*, 2001; Hampton *et al.*, 2008; Morales *et al.*, 2022).

Asymmetric F-actin organization around CCPs has also been linked to asymmetric pit closure (Shevchuk *et al.*, 2012; Yu and Yoshimura, 2023). In our observations, we localized CIP4, an established marker of asymmetric closure (Shevchuk *et al.*, 2012; Yu and Yoshimura, 2023), to apical CCPs in cultured epithelial cells. Additionally, CIP4 marked sites of microvillar growth. Asymmetric concentration of forces on the plasma membrane might provide a mechanical advantage that reduces the energy barrier to membrane fission and pit closure (Vasan *et al.*, 2020). How the unique physical properties that arise during these events (e.g., highly curved membrane or localized changes in membrane tension) contribute to microvillar formation remains unclear and should be the focus of future biophysical studies.

It is important to note that our data do not yet resolve whether F-actin asymmetry *per se* on CCPs is functionally required for microvillar initiation or simply reflects the predefined architecture of branched actin at these sites. Additionally, while our data do not allow us to determine whether the asymmetry in F-actin signal is a consequence of the early stages of microvillus core bundle formation, previous studies with nonpolarizing cells suggest this possibility is unlikely. Indeed, COS-7 and NIH-3T3 cells, which do not grow microvilli, still exhibit asymmetric F-actin accumulation at CCPs (Shevchuk *et al.*, 2012), indicating that F-actin asymmetry at these sites is not downstream of microvillar growth. Consistent with prior reports (Yu and Yoshimura, 2023), however, most F-actin-positive pits showed some degree of asymmetry, so the strongest predictor of microvillar formation was not the pattern of actin distribution, but rather the presence of an F-actin density itself.

In sum, our work provides evidence that actin-positive CCPs represent a major initiation platform for microvillar growth, with asymmetrically organized branched filament networks acting as the architectural precursor for protrusion formation. This model further resolves the relationship between endocytosis and microvillar biogenesis and suggests that the capacity to generate microvilli may arise broadly from pits that achieve the appropriate actin filament organization. Future studies should focus on delineating how these branched networks transition into linear bundles. Such insights may reveal general principles governing the formation of actin-based protrusions across diverse cellular contexts.

## MATERIALS AND METHODS

Request a protocol through *Bio-protocol*

### Cell culture, transfections, and drug treatment

LLC-PK1-CL4 (porcine kidney proximal tubule) cells were grown in 1X high glucose DMEM containing 2 mM L-glutamine (Corning #10-0130CV) supplemented with 10% fetal bovine serum (FBS; R&D Systems) and 1% L-Glutamine (Corning # 25-005-Cl). Cells were maintained in treated plastic culture flasks incubated at 37°C and 5% CO_2_, and tested for mycoplasma monthly using the MycoStrip Mycoplasma Detection Kit (Invivogen #rep-mys-50). LLC-PK1-CL4 cells stably expressing pmCherry-espin were previously generated using the protocol described in Gaeta *et al.*, 2021. For transient transfection, cells were seeded in 6-well plates and transfected at ∼90% confluency using Lipofectamine2000 with 3 µg DNA. After 4 hr of incubation with the lipid complex, cells were replated on CellVis 35 mm 1.5# glass bottom dishes for imaging the following day. In Arp2/3 inhibition experiments, CK666 was added to the media at a final concentration of 100 µM.

### Plasmid constructs

All constructs used in this study were described previously and/or obtained as noted in the Table 1.

### Live imaging microscopy

A Nikon Ti2 inverted light microscope equipped with a Yokogawa CSU-X1 spinning disk head, a Photometrics Prime 95B sCMOS camera, and 488 nm, 561 nm, and 647 nm excitation lasers. A 60x/1.49 NA or 100x/1.49 NA TIRF oil immersion objective was used for all live imaging experiments. Cells were maintained in a stage top incubator at 37°C with 5% CO2 (Tokai Hit). Z stacks of 5–8 µm using a 0.2–0.4 µm step size were collected using a triggered NIDAQ piezo Z stage.

### Immunofluorescence staining

***Tissue***. Mouse small intestinal tissue sections were deparaffinized and rehydrated through a descending ethanol series and subjected to antigen retrieval in a heated buffer at pH 9. Following cooling, sections were washed and blocked with 10% normal goat serum (NGS) and probed for γ-actin and ArpC2 in 1% NGS overnight at 4°C. The following day, sections were washed, incubated at room temperature with secondary antibodies, dehydrated through an ascending ethanol series, and mounted in ProLong Gold mounting medium.

***Cells*** Cells were fixed in 4% paraformaldehyde (PFA) at 37°C followed by permeabilization in 0.1% Triton X-100 in PBS. Following permeabilization, cells were blocked in 5% bovine serum albumin (BSA) at 37°C to reduce nonspecific antibody binding. Cells were then probed with primary antibody in 5% BSA overnight at 4°C. The following day, coverslips were washed and incubated at room temperature with secondary antibodies.

### Time series analysis of microvillus growth

Individual microvillar growth events were analyzed as previously described (Gaeta *et al.*, 2021). Briefly, a bounding ROI was manually drawn around the signal of interest, and the mean intensity was measured for each timeframe beginning one frame before the appearance of the fiduciary signal. Mean intensity values were normalized, with the smallest value equal to 0 and the largest equal to 1. Normalized values were then averaged and plotted with SEM. Growth events were selected using the *de novo* event criteria outlined in Gaeta *et al.* (2021).

### Spot detection and processing

TrackMate plugin (Ershov *et al.,* 2022) was executed via a custom Python for FIJI/ImageJ (Jython) script, which iteratively performed channel separation, Maximum Intensity Projection (MaxIP), and spot detection for each channel. A Difference of Gaussian (DoG) detector was applied with sub-pixel localization, median filtering, and an expected spot radius of *R*_*spot*_ of 0.15 µm was applied. Detection parameters were optimized for each channel based on imaging conditions and fluorescence intensity. Spots within 10 µm of the image margins were automatically excluded.

### Filament filtering and signal thresholding

A custom spatial anisotropy filter was developed to exclude linear F-actin and espin signals. For each spot, pixel intensities were sampled within an outer concentric annulus (inner radius: *R*_*spot*_; outer radius: 1.5*R_*spot*_). Average intensities were calculated across sliding angular sectors (bins) of 15° width and 2° step size. Bins were classified as “active” if their mean intensity exceeded 1.5 times the median of all bins. A spot was then considered part of a filament, and thus filtered out, if a contiguous arc of active bins corresponding to a length greater than 2.5 pixels was detected. To further exclude low-confidence detections and separate signal from background noise, dynamic intensity thresholds were established at the 3rd, 2nd, and 6th percentiles of the aggregated mean intensities for F-actin, espin, and CLTA, respectively. Lastly, puncta outside of the analyzed cell boundary were filtered out.

### Colocalization and grouping

Extracted x,y coordinates were used to calculate Euclidean distance vectors between all F-actin, espin, and CLTA puncta. Based on these measurements, only Espin spots within 0.25 µm of any F-actin were considered. Association groups were then formed based on the following rules that follow a hierarchical nearest-neighbor logic. 1. “minigroups” were created by assigning each F-actin punctum to a group. If the centroid Espin signal was within 0.2 µm of an F-actin punctum, it was assigned to the group. Resulting minigroups could then either have F-actin alone or F-actin and espin. 2. The nearest CLTA spot within 0.4 µm of the centroid of the F-actin signal from each minigroup was assigned to the group. 3. To prevent duplication, CLTA puncta could only be assigned to a single minigroup, determined by the closest proximity. To create polar plots (Figures 3 and 4), a separate Jython script located the CLTA position on the F-actin channel and calculated the mean pixel intensities over 360° in 10° bins within a 0.4 µm radius.

### Statistical analysis

All statistical testing was performed in GraphPad Prism 9 by running a Mann-Whitney test, a paired *t* test for pairwise comparisons, or a Kruskal-Wallis test to compare more than two conditions. For all figures, exact n values and their definitions are reported in the figure legends.

**TABLE 1:** Key resources used in this study.

Reagent or Resource	Source	Identifier
**Bacterial and viral strains**	
*E. coli* DH5-Alpha competent cells	Molecular Cell Biology Resource Core, Vanderbilt Medical Center	Item# DH5 Alpha
**Chemicals, peptides, and recombinant proteins**	
G418 Sulfate	Gold Biotechology	Catalogue# G-418-1
Lipofectamine 2000	Thermo Fischer Scientific	Catalogue# 11668019
Alexa Fluor 405 Phalloidin	Thermo Fischer Scientific	A22287
16% Paraformaldehyde	Electron Microscopy Sciences	Catalogue# 15710
ProLong Gold Antifade Reagent	Thermo Scientific	P36930
Triton X-100	Sigma	Catalogue# T8787
DMSO	Sigma	Catalogue# D8418
**Cell Lines**	
LLC-PK1-CL4	Gift from Dr. Carolyn Slayman (Yale University)	N/A
**Antibodies**	
anti-gamma actin (conjugated mouse) [1:200]	Santa Cruz Biotechnology	sc-65638
Anti-ArpC2 (rabbit) [11200]	Abcam	133315
Anti-CLTA	Proteintech	10852-1-AP
**Recombinant DNA**	
GFP-P16B	Gift from Dr. Dylan Burnette	
EGFP-Cortactin	Gift from Dr. Dylan Burnette	
EGFP-CIP4	Addgene	#178363
pmCherry-espin (rat)	Gift from Dr. James Bartles, NWU	N/A
**Software and Algorithms**	
FIJI		
NIS AR Elements Analysis	Nikon	N/A
Prism 9	Graphpad	N/A
BioRender	BioRender	N/A
Procreate	Savage Interactive	N/A
**Other**	
Mycostrip^TM^ – Myocplasma Detection Kit	InvivoGen	Catalogue# rep-mys50
35 mm #1.5 glass-bottom dishes	CellVis	Catalogue# D35-20-1.5N

## Supporting information





Supporting Video 1 Movie S1 Live cell movie of maximum intensity projection (MIP) confocal time series of 2 CL4 cells expressing (1) P16B and espin or (2) cortactin and espin. Time min: sec. Scale bar = 5 µm.

## Abbreviations used:

Arp2/3: actin related protein 2/3
CCP: clathrin coated pit
CIP4: cdc42 interactin protein 4
CL4: llc-PK1-CL4
CLTA: clathrin light chain A
CME: clathrin mediated endocytosis
EPS8: epidermal growth factor pathway substrate 8
IRTKS: insulin-receptor tyrosine kinase substrate
PI(4,5)P2: phosphatidylinositol 4-5 bisphosphate
TEM: transmission electron microscopy
TIRF: total internal reflection fluorescence.
