# Supplementary material for: Asymmetric branched F-actin networks at apical clathrin coated pits support microvillar biogenesis
Source: Mol Biol Cell. 2026 Apr 8;37(6):br18. doi: 10.1091/mbc.E25-12-0606 (PMC13200699; doi:10.1091/mbc.E25-12-0606)
Supplement: Supplementary file 1 [file mbc-37-br18-s001.pdf]

Supplemental Materials

Molecular Biology of the Cell

Perkins *et al*.

Asymmetric branched F-actin networks at apical clathrin coated pits support microvillar biogenesis

By Perkins et al.

SUPPLEMENTAL FIGURES

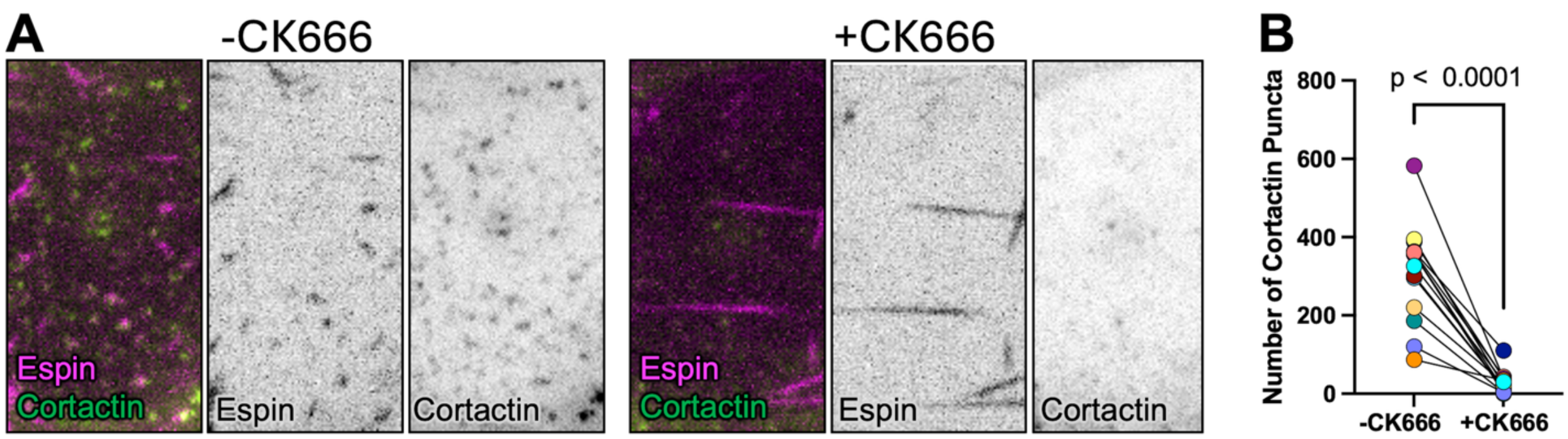

Figure S1. Microvilli grow from Arp2/3-generated branched F-actin networks. (A) Maximum intensity projection images (MIPs) of CL4 cells expressing espin and cortactin pre and post CK666 treatment. Cells were imaged before and after 45 min after incubation with 100 μM CK666. (B) Number of cortactin puncta before and after CK666 treatment. Each spot represents 1 cell.
